# A Scoping Review: The Effect of Anthropogenic Noise on Animal Welfare in Zoological Settings

**DOI:** 10.1002/vms3.71197

**Published:** 2026-08-31

**Authors:** Tamara N. Kruse, Mark Flint

**Affiliations:** ^1^ Dallas Zoo Dallas Texas USA; ^2^ Flint Welfcare Fredericktown Ohio USA

**Keywords:** acoustic, animal welfare, anthropogenic, noise, sound, zoo

## Abstract

With zoological institutions placing greater emphasis on improving animal welfare, there has been more research studying the impact of animal welfare, including correlation with increased visitors. Some studies focus on the total effect of visitor presence and behaviours impacted, whereas others highlight how noise from visitors and other events impacts animal behaviour and welfare. This scoping review was conducted to determine the impact of anthropogenic noise on animal welfare in zoological settings and identify knowledge gaps. Starting with an initial pool of 1608 articles, almost all publications in this review (*n* = 31) were published from 2014 to 2025, indicating the area is still a niche to be further explored. Most studies focused on mammalian species (*n* = 22, particularly non‐human primates), followed by avian species (*n* = 14) and reptiles (*n* = 3). Increased vigilance was the most common behaviour documented (*n* = 9). Thirteen publications suspected a negative welfare impact, while the remaining publications either found no substantial impact, could not determine impact, or did not address welfare directly. Fourteen publications recommended improving public education, sound barriers or increasing animals’ ability to choose a different location. Certain species and taxa, such as teleosts and reptiles in glass enclosures, lack representative studies on how anthropogenic noise affects their welfare. Further research that focuses on understanding the impact of noise on animal wellbeing could educate the public, allow restructure of habitats to accommodate animal needs and inform welfare assessments, which all can improve the welfare of animals in zoological settings.

## Graphical abstract

This scoping review was conducted to determine the impact of anthropogenic noise on animal welfare in zoological settings and identify knowledge gaps. Some studies found a negative impact with increased vigilance as a common behavioral change. A small number of studies evaluated physiological changes. Though other factors could be involved. Recommended improvements included providing more public education, other areas for animals to choose, and involving more detailed evaluation such as using soundscape assessments.

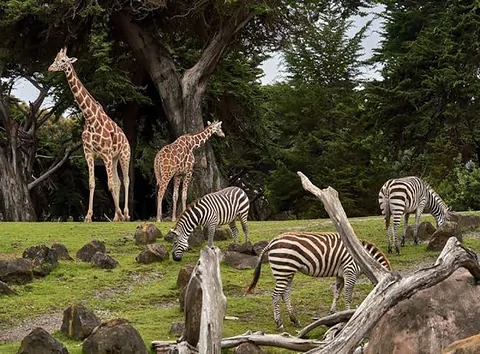

## 1 Introduction

Research on animal welfare in zoological settings has grown exponentially over the past decade as the public has become increasingly aware of its implications and importance to the health and wellbeing of animals within these settings (Badman‐King et al. 2024). The view of animal welfare at zoological facilities has become more complex, as it is not just about the aesthetics of ‘cages’, ‘bars’ and ‘concrete’ that define animal welfare. Large organisations representing zoological facilities and aquariums have created animal welfare guidelines for institutions to provide and maintain high standards of animal welfare (AZA 2024; BIAZA 2022; Harley and Clark 2019; ZAA 2024). Enclosure complexity, social groupings and cognitive enrichment are topics that are part of the guidelines for institutions to adhere to (AZA 2024). Research on these topics, along with how visitors affect animal behaviour has been published to continuously discover ways to improve animal welfare (Birke 2002; Fernandez et al. 2009). Multiple species and taxa have been investigated to determine how visitors affect animal welfare, with many focusing on intensity and numbers (Quadros et al. 2014). Recently, a publication investigated how noise from visitors and other events, such as concerts and construction, affects animal welfare. A survey of stakeholders was discussed, and articles were reviewed but did not go into detail about them (Rose and Rich 2025). This publication emphasised the importance of species and individual welfare assessments along with an animal's ability to control the response to the sounds in their environment.

Anthropogenic noise has been increasing over the years from increased infrastructure, transportation and overall human activity (Buxton et al. 2017). While human voices are thought of as the baseline definition, any human activity or human made objects can be considered as anthropogenic noise (Abramkina 2023). This can include everything from cell phone ringtones to engines and motors powering our movement and comfort. These articles focused on sound measured in decibels (dB), which can be used to create requirements for residents and businesses to follow to avoid causing noise disturbances in their neighbourhoods (Miedema et al. 2007). Although anthropogenic noise can disturb wildlife, animals could remove themselves from these areas (Shannon et al. 2016). However, animals in zoological settings may not have that opportunity, which increases the importance of investigating anthropogenic noise as a sustained and long‐term contributor influencing animal welfare. Not all changes have to be drastic to improve animal welfare, as events and construction are necessary to improve structures and generate funds to continuously support the zoological institution.

Continuous monitoring of the animal's welfare is important to prevent or lessen chronic negative behavioural issues (Orban et al. 2017; Liu et al. 2017). This scoping review focuses on how anthropogenic noise from visitors or events caused by humans (concerts or construction) affects animal welfare in a zoological setting. The results of this investigation could lead to changes in event location, sound pressure levels or duration, implementation of noise‐dampening structures or educating the public about lowering their voices.

## 2 Materials and Methods

### 2.1 Initial Literature Search

An initial literature search was performed by a single investigator in April 2026 using the PubMed and Google Scholar databases and searching for cited literature throughout articles in the final review. All available documents on each of the search terms, which included a combination of the words ‘noise’, ‘zoo’, ‘visitor’, ‘animal’ and ‘welfare’. The search included English‐only publications with no limitations on publication year and initially accounted for all types of documents (e.g., journal articles, reviews, theses and book chapters). Furthermore, the location of the study was not excluded, as the overarching concept could be in any location. The databases were last accessed on 24, April 2026. In the literature search, only documents involving the following concepts were used. The first concept was that animals were studied only in zoological settings. The second concept involved sound, noise or acoustics of anthropogenic origin. The third concept limited the review to studies that only investigated animal welfare. Each concept was combined in a single search with changes to the keywords (i.e., noise vs. sound vs. acoustic) to capture as much pertinent literature available.

The first literature search was conducted in the ‘PubMed’ database using a combination of keywords. The second search was conducted in the ‘Google Scholar’ database using the keywords anthropogenic noise + zoo + animal welfare, and only review articles were considered, as any type of article would bring up too many false results. An additional literature search was conducted by reviewing the titles and abstracts of the cited literature from the review articles that passed the eligibility criteria and screening.

### 2.2 Eligibility Criteria and Screening

Publications obtained from the literature search were imported into Mendeley (Mendeley Reference Manager, version 2.144.0, Elsevier, Amsterdam, The Netherlands) to systematically select relevant papers. The eligibility and selection criteria for final articles were conducted by the same investigator using a modified version of PRISMA 2020 guidelines for a more complete report while adjusting for limited research staff and time (Page et al. 2021). First, all duplicates were excluded from the study. Second, the title and abstract of each publication were evaluated according to the following exclusion criteria: (1) not involving visitor noise affecting animal welfare in zoo animals; (2) not concerning animal welfare and anthropogenic noise at zoological facilities (i.e., publications regarding anthropogenic noise affecting animals in nature/lab/sanctuaries/research centres were excluded); (3) not concerning aspects or measurements involving animal welfare or anthropogenic noise such as behaviour, glucocorticoids, decibels, frequency, sound level or acoustic environment; (4) use of technology to measure acoustics or sound in zoological settings but not to assess animal welfare; (5) was not a full article (e.g., conference abstract/paper); and (6) not a peer‐reviewed publication. After reviewing and eliminating the abstracts based on the second criteria, the full text of the remaining publications was extracted for an in‐depth screening. These publications were the final articles included in the scoping review (Table S1).

For each publication, the following initial information was noted: (1) title; (2) journal; (3) year of publication; (4) animal species studied; (5) country where the study was conducted; and (6) the type of parameters evaluated (i.e., behavioural, physiological or both). Other topics that were reviewed included (1) the types of behaviours documented, (2) association or lack of association between the behaviour and increased anthropogenic noise or acoustics, (3) other variables that could have contributed to the behavioural change observed, (4) whether welfare was impacted and (5) types of recommendations to mitigate noise or acoustic aversion.

## 3 Results

### 3.1 Literature and Search Strategy

The initial literature search strategy yielded 1608 publications (*n* = 691 from ‘PubMed’, n = 917 from Google Scholar). Of these, 601 publications were duplicates, and another 902 publications were eliminated as the title did not adhere to the appropriate criteria. The abstracts of the 105 remaining publications were screened for eligibility. An additional 11 publications, identified from the reference lists of selected publications, were evaluated for relevance. After the final screening of the full publications, 34 peer‐reviewed publications met criteria for inclusion in this scoping review. Of these publications, 31 were research studies and three were case studies.

### 3.2 Publication Characteristics

**FIGURE 1:**
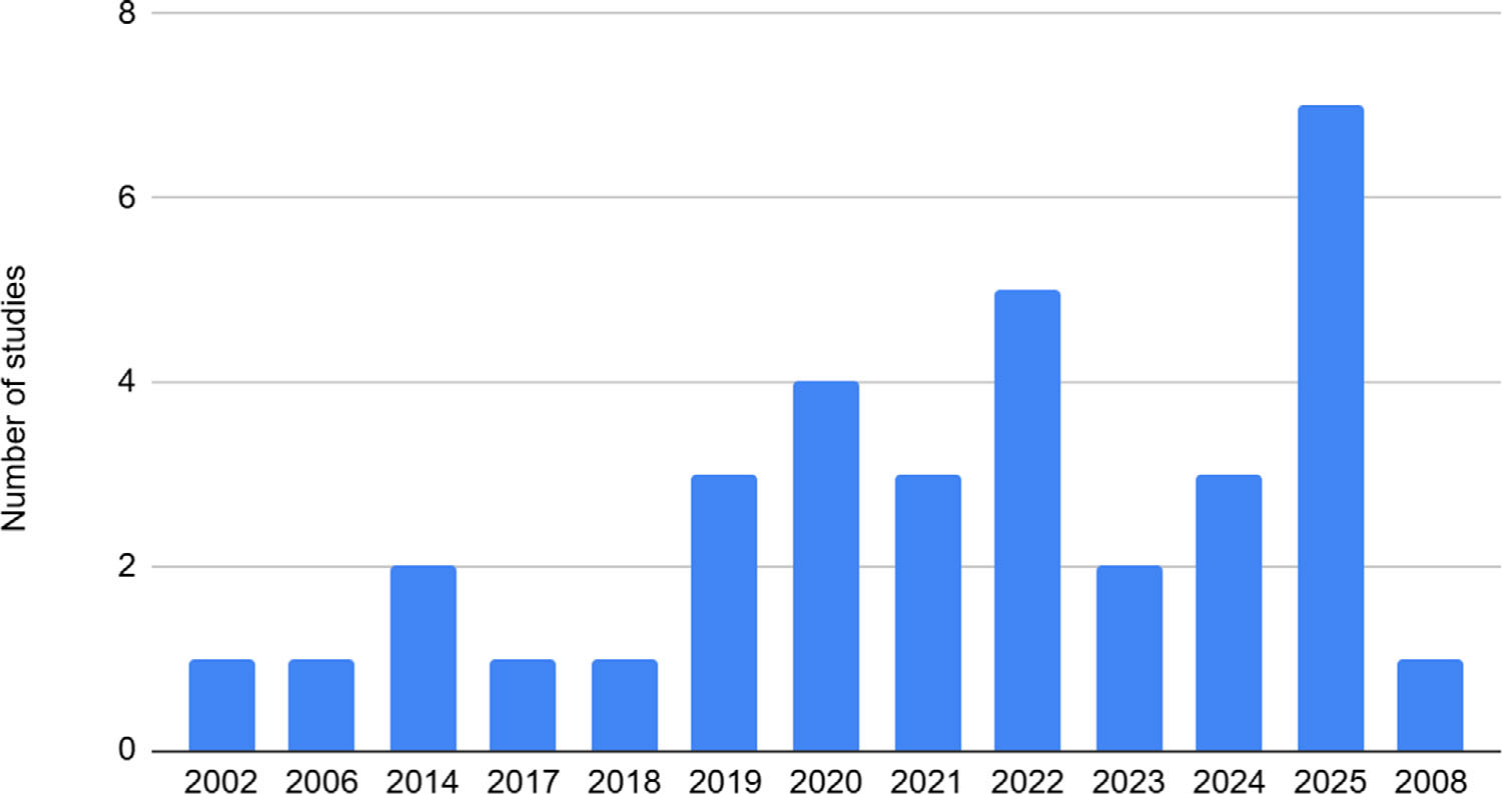
Temporal trend of studies in journals focusing on anthropogenic noise and effects on animal welfare in zoological settings.

The topic of anthropogenic noise impacting animal behaviour and welfare could be considered relatively new because almost all publications were conducted between 2014 and 2025 (*n* = 31). Figure 1 highlights the temporal trend of studies reviewed in this manuscript. Though no single country predominated in the publication of this topic, as seen in Table 1, the studies from the United Kingdom (UK) and the United States (US) collectively account for more than half of them in this review (*n* = 22, 64.7%). Even though the majority of species studied were from mammalian taxa (*n* = 22) (Table 2), with non‐human primates being the most common (*n* = 10), there were publications involving avian species (*n* = 13), and reptiles (an American alligator, a Siamese crocodilian and one of a variety of semi‐aquatic turtles) (*n* = 3). There were more individual avians evaluated than mammals. Half of the studies (*n* = 17) evaluated two or fewer individual animals (Table 2). Two studies focused on a large variety of aviary species housed in free‐flighted aviaries (Blanchett et al. 2020; Rose et al. 2022). Three studies did not focus on a specific taxa and focused more on the location of animals in proximity to anthropogenic noise (Harley et al. 2022; Monticelli et al. 2022; Salma et al. 2024). No studies evaluated teleost species.

**TABLE 1:** Country of origin for research study of anthropogenic noise and effects on animal welfare in zoological settings.

Country	Number of studies, % of total	Author and year of study
United Kingdom	13, 38.2%	Dancer and Burns 2019
		de Queiroz et al. 2025 Gray et al. 2024
		Harley et al. 2019
		Harley et al. 2022
		Hashmi and Sullivan 2020
		Powell et al. 2006
		Queiroz et al. 2025
		de Abreu Rezende et al. 2023
		Rose et al. 2021
		Rose et al. 2022
		Sulser et al. 2008
		Williams et al. 2021
United States	9, 26.5%	Birke 2022
		Blanchett et al. 2020
		Cronin et al. 2018
		Edes et al. 2021
		Readyhough et al. 2022
		Roth and Cords 2020
		Thomson and O'Brien 2025
		Wark et al. 2023
		Westcott et al. 2025
Australia	3, 8.8%	Fanning et al. 2020
		Li et al. 2025
		Sherwen et al. 2014
Brazil	3, 8.8%	Marques et al. 2025
		Monticelli et al. 2022
		Quadros et al. 2014
New Zealand	2, 5.9%	Davison et al. 2022
		Jakob‐Hoff et al. 2019
China	1, 2.9%	Liu et al. 2017
Pakistan	1, 2.9%	Salma et al. 2024
Portugal	1, 2.9%	Cunha and Wasterlain 2025
Singapore	1, 2.9%	Tay et al. 2025

**TABLE 2:** Species evaluated for behavioural change from changes in anthropogenic noise.

Species	Number of studies	Number of sample size	Study
Mammals	28	141	
*Capuchin*	1	4	Cunha and Wasterlain 2025
*Cheetah*	1	2	Westcott et al. 2025
*Chimpanzees*	1	4	Cronin et al. 2018
*Elephant (Asian)*	1	2	Jakob‐Hoff et al. 2019
*Fox (crab‐eating)*	1	6	Marques et al. 2025
*Giant panda*	2	2 7	Powell et al. 2006 Liu et al. 2017
*Gibbon (pleated)*	1	4	Hashmi and Sullivan 2020
*Gibbon (white‐handed)*	1	2	Cunha and Wasterlain 2025
*Giraffe*	2	2 3	Jakob‐Hoff et al. 2019 Westcott et al. 2025
*Gorilla*	2	2 6	Cronin et al. 2018 Hashmi and Sullivan 2020
*Langur (Ebony)*	1	6	Roth and Cords 2020
*Lion (Asiatic)*	1	3	Williams et al. 2021
*Macaque (Japanese)*	1	4	Cronin et al. 2018
*Macaque (Sulawesi)*	1	55	Dancer and Burn 2019
*Meerkat*	1	10	Sherwen et al. 2014
*Okapi*	1	1	de Queiroz et al. 2025
*Orangutan*	3	5	Birke 2002
		5	Hashmi and Sullivan 2020
		4	Queiroz et al. 2025
*Peccary (collared)*	1	3	Fanning et al. 2020
*Sloth (Linneau's Two‐Toed)*	1	2	de Abreu Rezende et al. 2023
*Snow leopard*	1	3	Sulser et al. 2008
*Tamarin (pied)*	1	2	Wark et al. 2023
*Tiger (amur)*	1	2	Harley et al. 2019
Avian	14	197	
*Emu*	1	2	Jakob‐Hoff et al. 2019
*Flamingo (Chilean)*	1	53	Rose et al. 2021
*Flamingo (greater)*	1	56	Rose et al. 2021
*Hornbill (black‐casqued)*	1	2	Tay et al. 2025
*Hornbill (crowned)*	1	2	Tay et al. 2025
*Hornbill (rhino)*	1	2	Tay et al. 2025
*Hornbill (wreathed)*	1	2	Tay et al. 2025
*Hornbill (greater Indian)*	2	2	Readyhough et al. 2022
		1	Tay et al. 2025
*Kiwi*	1	15	Davison et al. 2022
*Penguin (Fjordland)*	1	2	Fanning et al. 2020
*Penguin (gentoo)*	1	14	Edes et al. 2021
*Penguin (king)*	1	20	Edes et al. 2021
*Penguin (southern Rockhopper)*	1	24	Edes et al. 2021

### 3.3 Behavioural Assessment

**FIGURE 2:**
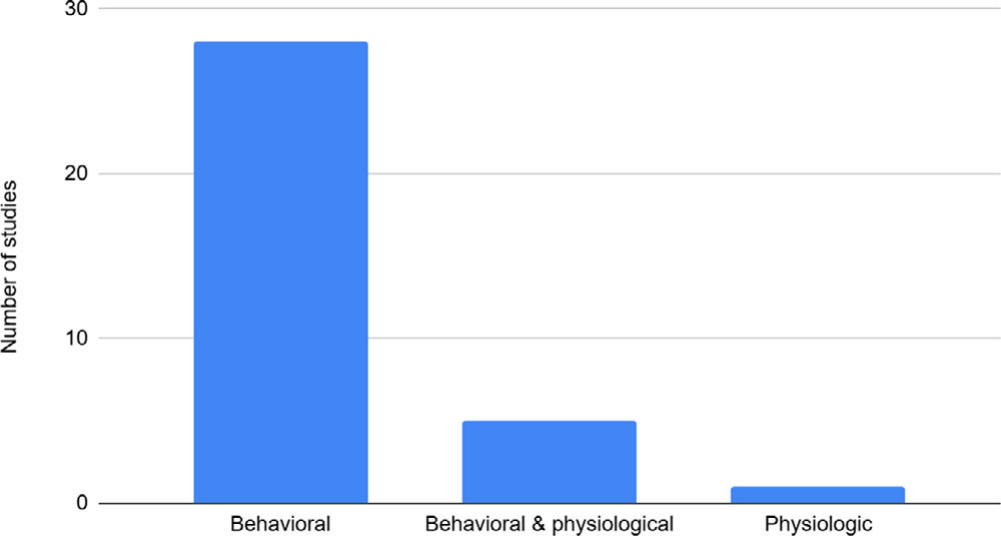
Number of studies focusing on behavioural and/or physiological changes secondary to anthropogenic noise in zoological settings.

Behavioural change associated with anthropogenic noise was the primary measure in this scoping review (*n* = 28). Ethograms were performed in all research studies observing behaviours with detailed descriptions and multiple categories respective to the species observed. Figure 2 demonstrates the number of studies focusing on behavioural and/or physiological changes. Eight publications using video footage noted limitations related to missing footage or animals moving outside the camera frame (Cronin et al. 2018; de Queiroz et al. 2025; Fanning et al. 2020; Harley et al. 2022; Jakob‐Hoff et al. 2019; Marques et al. 2025; Monticelli et al. 2022; Rose et al. 2022). Any behavioural changes during these studies are documented in Table 3 with increased vigilance being the most common behaviour documented (*n* = 10) (Cunha and Wasterlain 2025; Cronin et al. 2018; Dancer and Burn 2019; Fanning et al. 2020; Hashmi and Sullivan 2020; Jakob‐Hoff et al. 2019; Quadros et al. 2014; Rose et al. 2022; Tay et al. 2025; Westcott et al. 2025). Six publications reported no statistically significant or substantial behavioural change associated with increased anthropogenic noise (Harley et al. 2019; Liu et al. 2017; Queiroz et al. 2025; Readyhough et al. 2022; Salma et al. 2024; Sherwen et al. 2014). Though abnormal behaviours were documented, none of the articles stated that health was strongly affected or described overt abnormal behaviours such as self‐inflicted trauma or substantial aggression towards other animals.

**TABLE 3:** Behavioural and physiologic change observed during increased anthropogenic noise in zoological settings.

Behaviour outcome and direction of effect (*n* of studies)	Species	Noise source	Glucocorticoid changes	Study
Increased vigilance (10)	Macaques Gibbon & capuchin Macaques Peccaries (collared) Orangutan Giraffe Multiple taxa Aviary species Hornbill species Cheetah	Events Guided tour Visitors Concert Visitors Construction Visitors Visitors Visitors Concert	No changes	Cronin et al. 2018 Cunha and Wasterlain 2025 Dancer and Burn 2019 Fanning et al. 2020 Hashmi et al. 2020 Jakob‐Hoff et al. 2019 Quadros et al. 2014 Rose et al. 2022 Tay et al. 2025 Westcott et al. 2025
Increase distance from visitors or being out of sight (5)	Aviary species Multiple taxa Linneau's sloth Aquatic turtles Tamarin (pied)	Visitors Visitors Visitors Visitors Visitors	No changes	Blanchett et al. 2020 Harley et al. 2022 de Abreu Rezende et al. 2023 Thomson et al. 2025 Wark et al. 2023
Decreased rest (4)	Multiple taxa Crab‐eating fox Giant panda Lion (Asiatic)	Visitors Visitors Construction Construction	Increased	Harley et al. 2022 Marques et al. 2025 Powell et al. 2006 Williams et al. 2021
Increased self or social grooming (3)	Giraffe Giant panda Langur (Ebony)	Construction Construction Visitors	Increased	Jakob‐Hoff et al. 2019 Powell et al. 2006 Roth and Cords 2020
Increased Pacing (3)	Kiwi Tiger Lion (Asiatic)	Visitors Visitors Construction		Davison et al. 2022 Li et al. 2025 Williams et al. 2021
Decreased appetite (2)	Okapi Gorilla	Environment/visitor Visitors	Increased	de Queiroz et al. 2025 Hashmi et al. 2020
Increased locomotion but not considered pacing (2)	Giraffe, Emu Chilean flamingo	Construction Visitors	No changes	Jakob‐Hoff et al. 2019 Rose et al. 2021
Increased swimming (2)	Fjordland Penguin Multiple penguins	Concert Visitors		Fanning et al. 2020 Edes et al. 2021
Change in circadian activity (increased during day) (1)	Multiple taxa	Visitors, events, environmental		Monticelli et al. 2022
Increased time underwater (1)	Siamese crocodile	Visitors		Gray et al. 2024
Increased protectiveness of infants (holding more often) (1)	Orangutan	Visitors		Birke 2002
Increased rest (1)	Snow leopard	Construction		Sulser et al. 2008
NA	Orangutan	Visitors	Increased	Queiroz et al. 2025

### 3.4 Physiologic Assessment

A different parameter documented to help assess the impact of anthropogenic noise in a zoological setting included the measurement of glucocorticoid (GC) concentrations (*n* = 6) (Table 3). Five publications measured GC levels along with behavioural observation (de Queiroz et al. 2025; Jakob‐Hoff et al. 2019; Powell et al. 2006; Wark et al. 2023; Westcott et al. 2025) and one of them as a sole indicator (Queiroz et al. 2025). The majority of them collected and evaluated GC in faecal samples (*n* = 5) (de Queiroz et al. 2025; Jakob‐Hoff et al. 2019; Queiroz et al. 2025; Wark et al. 2023; Westcott et al. 2025), and the remaining publication evaluated GC concentrations from urine samples (Powell et al. 2006). Three of the six reported elevated GC concentrations: one attributed to increased visitor presence (in okapis) (de Queiroz et al. 2025), one to ventilation system noise (in orangutans) (Queiroz et al. 2025) and one to normal physiological variation unrelated to noise (in a giant panda) (Powell et al. 2006). The remaining three publications found no elevation or no correlation between GC levels and behavioural change (Jakob‐Hoff et al. 2019; Wark et al. 2023; Westcott et al. 2025). No publication incorporated additional physiological or health‐based welfare indicators.

### 3.5 Sources of Anthropogenic Noise Prioritised in the Literature

In this scoping review, the publications focused on specific origins of anthropogenic noise, with the majority of them evaluating the impact of visitors on behavioural or physiological changes (*n* = 27). The other studies evaluated construction noise (*n* = 5) (Harley et al. 2019; Jakob‐Hoff et al. 2019; Powell et al. 2006; Sulser et al. 2008; Williams et al. 2021) and concerts (*n* = 2) (Fanning et al. 2020; Harley et al. 2022). Though visitor noise was the initial evaluation, two publications suspected that the ventilation system was the actual cause for behavioural changes, as the decibels did not significantly change with an increase in the number of visitors (de Queiroz et al. 2025; Queiroz et al. 2025). A third publication found that behavioural changes could have been more associated with the waterfall feature in the habitat rather than with visitor noise (Wark et al. 2023). Even though the focus of the publications was on the impact of anthropogenic noise, some publications suggested that environmental acoustics and habitat design (*n* = 8), termed as soundscape, contributed to behavioural change (Monticelli et al. 2022; Queiroz et al. 2025; Rose et al. 2021; Rose et al. 2022; Salma et al. 2024; Tay et al. 2025; Wark et al. 2023; Westcott et al. 2025). One study found that their results suggested reducing visitor numbers or non‐visiting hours did not guarantee a quieter environment and emphasised the persistent impact of mechanical infrastructure on zoo soundscapes (de Queiroz et al. 2025). Though soundscapes could lead to larger impacts, they could also help mitigate them, as a separate study found that the enclosure's soundscape largely buffered against short‐term fluctuations in external noise (Queiroz et al. 2025). Additionally, several publications noted that not all behavioural changes were attributable solely to anthropogenic noise and instead associated with visual stimuli (i.e., increased number of visitors or being able to see construction workers) (Cunha and Wasterlain 2025; Fanning et al. 2020; Harley et al. 2019; Harley et al. 2022; Westcott et al. 2025) or suspected individual or species variance (Blanchett et al. 2020; Harley et al. 2019; Harley et al. 2022; Hashmi et al. 2020; Jakob‐Hoff et al. 2019; Quadros et al. 2014; Sherwen et al. 2014; Williams et al. 2021), where some were more sensitive to noise than others (Cunha and Wasterlain 2025; de Abreu Rezende et al. 2023; Harley et al. 2019; Harley et al. 2022; Rose et al. 2021; Salma et al. 2024; Williams et al. 2021).

### 3.6 Objective Sound Measurement

A sound meter was the most common tool used to measure dB levels. Sound meters measuring anthropogenic noise levels were positioned on the public‐facing side of the habitat. Sound pressure levels ranged between 51.5 and 122.2 dB, with the highest noise levels recorded near a sloth habitat in a walk‐through habitat (de Abreu Rezende et al. 2023). Concert noise reached a maximum of 88 dB and construction noise a maximum of 76 dB. One publication documented the acoustic characteristics between construction and non‐construction days (average 55 dB) with different types of noise measured in hertz (Hz) (Powell et al. 2006). Another compared how different frequency weighting‐filters (dBA and dBC) could affect different penguin species (gentoo, king and southern rockhopper). Only southern rockhoppers demonstrated a positive effect of pool use with elevated dBC levels but no significant effect with dBA levels (Edes et al. 2021).

### 3.7 Welfare Outcomes

After reviewing the selected publications, 11 of them suspected a negative welfare impact associated with anthropogenic noise, based on behavioural changes observed during the study period (Birke 2002; Davison et al. 2022; de Abreu Rezende et al. 2023; de Queiroz et al. 2025; Monticelli et al. 2022; Queiroz et al. 2025; Rose et al. 2022; Sulser et al. 2008; Tay et al. 2025; Westcott et al. 2025; Williams et al. 2021). One of the studies suspected a negative impact associated with increased noise from visitors because the offspring exhibited behaviours that could correlate to increased stress (Birke 2002). Nine publications were unable to determine whether welfare was affected (Cunha and Wasterlain 2025; Dancer and Burn 2019; Fanning et al. 2020; Gray et al. 2024; Hashmi and Sullivan 2020; Li et al. 2025; Marques et al. 2025; Roth and Cords 2020; Sherwen et al. 2014), seven publications reported no substantial welfare impact (Blanchett et al. 2020; Edes et al. 2021; Jakob‐Hoff et al. 2019; Powell et al. 2006; Quadros et al. 2014; Readyhough et al. 2022; Thomson and O'Brien 2025), and seven did not address welfare as an outcome (Cronin et al. 2018; Harley et al. 2019; Harley et al. 2022; Liu et al. 2017; Rose et al. 2021; Salma et al. 2024; Wark et al. 2023). No publication reported evidence of significant health effects or overt abnormal behaviour, such as self‐inflicted trauma or substantial aggression, associated with anthropogenic noise exposure. One study documented that though no substantial behavioural changes were noted at a group level, it did not exclude any negative impacts on welfare from visitors or noise pollution (Quadros et al. 2014). Welfare assessment protocols were not consistently described across publications, and several authors recommended individualised, species‐specific assessment approaches going forward (Fanning et al. 2020; Harley et al. 2022; Rose et al. 2021; Salma et al. 2024; Williams et al. 2021).

### 3.8 Recommendations

Fourteen publications offered recommendations for mitigating anthropogenic noise, six of which specifically addressed strategies for improving animal welfare (Table 4). The most common recommendation was increased public education, which was noted in all six publications (Blanchett et al. 2020; Dancer and Burn 2019; de Queiroz et al. 2025; Gray et al. 2024; Hashmi and Sullivan 2020; Quadros et al. 2014; Thomson and O'Brien 2025). The second most common recommendation was to improve or increase an animal's choice and control of its location in the habitat (*n* = 4) (Birke 2002; Blanchett et al. 2020; Jakob‐Hoff et al. 2019; Salma et al. 2024). Collectively, these findings indicate that the literature has prioritised behavioural documentation of visitor‐driven noise, while objective acoustic measurement, physiological assessment and direct welfare characterisation remain comparatively underrepresented.

**TABLE 4:** Recommendations to decrease anthropogenic noise or improve animal welfare from increased noise.

**Recommendation**	**Number of studies**	**Author and year of study**
Public education (including signage)	7	Blanchett et al. 2020 Dancer and Burn 2019 de Queiroz et al. 2025 Gray et al. 2024 Hashmi and Sullivan 2020 Quadros et al. 2014 Thomson and O'Brien 2025
Improving or increasing animal's choice and control	4	Birke 2002 Blanchett et al. 2020 Jakob‐Hoff et al. 2019 Salma et al. 2024
Increase control of visitor numbers and visiting times	3	Gray et al. 2024 de Queiroz et al. 2025 Rezende et al. 2023
Habituation or adding soothing background noise	2	Blanchett et al. 2020 Davison et al. 2022
Increase vegetation/foliage within habitat	2	de Queiroz et al. 2025 Thomson and O'Brien 2025
Change enclosure design to make it quieter	2	de Queiroz et al. 2025 Thomson and O'Brien 2025
Continue monitoring animal's behaviour	2	de Queiroz et al. 2025 Williams et al. 2021

## 4 Discussion

Based on this scoping review, anthropogenic noise and its impact on animal behaviour in zoological settings appear to be an emerging topic, with growing interest documented across institutions, species and geographic regions. While publications trended toward behavioural observations associated with visitor noise, construction and event‐related noise were also represented in this scoping review and are relevant considerations as zoological institutions increasingly rely on renovations and ticketed events to support operations. Though the overall impact on animal welfare, soundscape‐level acoustic assessment and noise mitigation recommendations were not widely published, these factors provide opportunities to extend the knowledge of existing work.

### 4.1 Behaviour Assessment

Behavioural observation was the most consistently applied method in this scoping review, with ethograms serving as the predominant assessment tool. Ethograms allow researchers to tailor monitoring to species‐specific behaviour, standardising observation and reducing variability between observers (Curran et al. 2016; Fanning et al. 2020). Under the Five Domains model, an animal modifying its behaviour in response to an environmental stressor reflects the functional domain of behavioural interaction; when that behaviour is repeated or sustained, it may also extend into the mental or affective state domain (Mellor et al. 2020). Increased vigilance was the most frequently documented behavioural response in this review. Vigilance is described as monitoring the environment for threats such as predators or social rivals, often characterised by scanning behaviours like lifting the head while foraging (Campos et al. 2017); several publications in this review extended this interpretation to include visitor avoidance specifically. The consistent application of ethograms across this literature has established a solid behavioural foundation, which, when paired with the additional acoustic measurement, could be extended toward a fuller characterisation of welfare impact rather than behavioural change alone.

### 4.2 Physiologic Assessment

A smaller subset of publications incorporated physiological measurement, most commonly glucocorticoid (GC) concentration, as a complementary or, in one case, primary indicator of stress. Measuring GC levels offers an accessible, non‐invasive window into hypothalamic‐pituitary‐adrenal (HPA) axis activity and has long been a standard tool in zoo‐based welfare research (Brown 2023; Davis 2009; Learmonth and Hemsworth 2024; Scherpenhuizen 2022; Steinman et al. 2020). However, a substantial body of more recent literature has debated the interpretive value of GC measurement as a stand‐alone welfare indicator (Ralph and Tilbrook 2016; Romero and Beattie 2022; Romano et al. 2010; Tiemann et al. 2023). Findings within this scoping review align with previous research indicating that GC elevation could be influenced by factors unrelated to noise exposure, including reproductive status, sample matrix and circadian variation (Ralph and Tilbrook 2016; Romero and Beattie 2022; Romano et al. 2010; Tiemann et al. 2023). In context of this review, these findings are consistent with the broader literature's reconsideration of GC as a stand‐alone welfare measure; rather than diminishing the contribution of these six studies, this context suggests that pairing GC measurement with complementary indicators, such as the cognitive bias testing already employed in one publication in this review, could meaningfully strengthen future welfare conclusions (Cronin et al. 2018). Such approaches would shift the field toward the multidimensional welfare assessment frameworks increasingly recommended in the broader zoo animal welfare literature.

### 4.3 Soundscapes

The relatively limited use of soundscape as a conceptual framework in this scoping review (and beyond) represents a clear opportunity to deepen future investigations of anthropogenic noise and welfare. Soundscape refers to the full acoustic environment of a habitat, encompassing not only anthropogenic noise but also natural sounds such as rustling leaves and wildlife vocalisations, as well as how habitat design shapes the way sound travels and is experienced from an animal's perspective (de Abreu Rezende et al. 2023; Orban et al. 2017; Pelletier et al. 2020). One of the earliest examples of this approach in the zoo literature was a case study involving a giant anteater. Although it did not use the term ‘soundscape’ explicitly, this article evaluated environmental acoustics and associated behavioural changes (Orban et al. 2017). That study has since served as a model for subsequent soundscape‐focused research.

Incorporating soundscape assessment into future research could allow investigators to evaluate anthropogenic noise within the broader acoustic context an animal actually experiences, rather than as an isolated variable. This shift in framing could meaningfully strengthen the field's capacity to distinguish noise‐specific welfare effects from the influence of a habitat's overall acoustic design. In practice, soundscape evaluation may require only modest adjustments for some habitats, while others may benefit from structural interventions such as sound‐dampening materials to mitigate noise from visitors, ventilation systems, or other sources (Li 2011; Orban et al. 2017).

### 4.4 Welfare Impacted by Noise

Though behavioural observation is itself a recognised aspect of animal welfare assessment, most included publications evaluated a single measure of welfare rather than employing comprehensive, multidimensional assessments. Fifteen publications explicitly characterised welfare impact, while the remainder focused predominantly on behavioural change without integrating additional welfare dimensions such as physiological or cognitive indicators (Birke 2002; Blanchett et al. 2020; de Abreu Rezende et al. 2023; de Queiroz et al. 2025; Davison et al. 2022; Edes et al. 2021; Jakob‐Hoff et al. 2019; Monticelli et al. 2022; Queiroz et al. 2025; Rose et al. 2022; Sulser et al. 2008; Tay et al. 2025; Thomson and O'Brien 2025; Westcott et al. 2025; Williams et al. 2021). Some publications, while not directly characterizing welfare outcomes, identified the need for improved, standardised assessment protocols going forward (Orban et al. 2017; Romain et al. 2026; Wolfensohn et al. 2018). Accrediting associations for zoological institutions emphasise and require routine animal welfare assessments; however, welfare assessment protocols can vary considerably across institutions depending on their collection, habitat designs and staff availability (AZA 2024; BIAZA 2022; ZAA 2024). The absence of standardised noise‐specific welfare assessment protocols across zoological institutions represents a meaningful gap, as the welfare significance of documented behavioural changes may be difficult to interpret without complementary physiological or cognitive measures. Many publications indicated multiple variables that could overshadow the impact from solely anthropogenic noise such as visitor presence, species or individual sensitivity and reproductive hormonal changes (Birke 2002; Blanchett et al. 2020; Dancer and Burn 2019; Harley et al. 2019; Hashmi and Sullivan 2020; Liu et al. 2017; Owen et al. 2004; Readyhough et al. 2022; Roth and Cords 2020; Thomson and O'Brien 2025). Other publications suspected species or individual sensitivity or other noises that could have contributed, such as precipitation‐related acoustic interference, making it challenging to discern welfare impact (Davison et al. 2022; de Abreu Rezende et al. 2023; de Queiroz et al. 2025; Powell et al. 2006; Rose et al. 2021; Queiroz et al. 2025; Tay et al. 2025; Westcott et al. 2025; Williams et al. 2021). Each of these factors complicates efforts to isolate anthropogenic noise as the specific cause of observed welfare effects. Additional areas of future investigation include understanding the impact of banging on enclosure glass structures and precise reasons for the behavioural change (i.e., noise levels, act itself and proximity of the visitor to the animal); how animals react to different frequencies created by humans; whether animals respond differently to chronic versus acute noise exposure; and evaluating changes in other noise‐sensitive species to increased anthropogenic noise (Edes et al. 2021; Gray et al. 2024; Sherwen et al. 2014). Collectively, these confounding factors underscore why welfare characterisation is less documented than behavioural observations in this field.

Under the Five Domains model, each domain contributes cumulative positive or negative inputs that collectively determine an animal's overall mental state, rather than operating as competing priorities (Mellor et al. 2020). Anthropogenic noise primarily affects the physical environment domain, but its influence on behavioural interaction and affective state must be considered within the context of the animal's broader welfare profile. The ventilation system findings illustrate this complexity, as mechanical noise may negatively affect the physical environment domain, but removing or reducing ventilation could compromise thermal comfort and air quality, resulting in a net negative welfare outcome across domains (de Queiroz et al. 2025; Queiroz et al. 2025). This example underscores that evaluating the welfare impact of anthropogenic noise cannot be done in isolation from other domains it intersects with.

### 4.5 Noise‐Friendly Zoological Settings

Recommendations for mitigating anthropogenic noise represented a smaller but valuable portion of the included literature, clustering around three primary strategies: public education, environmental choice and construction or operational planning. Educating visitors through signage or trained personnel was among the most frequently cited recommendations, with one study finding that signage made a measurable difference in observed noise levels. This approach may carry greater weight given growing public awareness of animal welfare considerations in zoological settings (Dancer and Burn 2019; Miller 2012). A second prominent recommendation centred on providing animals the ability to choose alternative locations within their habitat. Several publications documented animals retreating to indoor or alternative spaces in response to noise aversion (Harley et al. 2022; Steinbrecher et al. 2023; Sulser et al. 2008; Wark et al. 2023). In previous literature, the opportunity for an animal to have control and a choice of location in a habitat has been identified as an important contributor to positive welfare (Ritzler et al. 2021). For many species, control over one's environment is itself a meaningful welfare factor, which suggests that a flexible habitat design for animals with low‐noise retreat spaces could benefit a wide range of taxa.

Awareness of species‐specific behavioural responses can also inform operational decisions, allowing zoological leadership and animal care staff to implement modifications that preserve visitor engagement while supporting animal welfare, such as relocating more sensitive species during extended construction periods. Training and habituation approaches have also shown promise in several studies as a means of reducing noise‐related behavioural disturbances (Cronin et al. 2018; Quadros et al. 2014; Rose and Rich 2025), suggesting institutions may benefit from incorporating these approaches more systematically into noise mitigation planning.

## 5 Conclusions

Anthropogenic noise alone may not cause negative impacts on welfare, as some of the studies could not separate noise from visual disturbances. Species and individuals may have different responses to visitors’ presence and noise, which emphasises the importance of individual welfare assessments. Individual welfare assessments could allow staff and institutions to prepare and plan more appropriately. Early and individualised assessment may prevent costly habitat reconstruction by identifying minor, targeted modifications before animal welfare is substantially compromised.

While measuring sound pressure levels is helpful, it has limitations and may not accurately represent the level the animal is experiencing. Further research is warranted, including the incorporation of noise disturbances into welfare assessment tools and whether certain behaviours are secondary to anthropogenic noise or are considered negative. As zoo accreditation standards evolve, noise may warrant more formal inclusion in welfare assessment frameworks.

## Author Contributions

**Tamara N. Kruse**: conceptualisation, investigation, methodology, resources, data curation, validation, visualisation, writing – original draft. **Mark Flint**: supervision, writing – review and editing.

## Funding

The authors have nothing to report.

## Ethics Statement

The authors confirm that the ethical policies of the journal, as noted on the journal's author guidelines page, have been adhered to. No ethical approval was required as this is a review article with no original research data

## Conflicts of Interest

The authors declare no conflicts of interest.

## Supporting information




**Supporting File**: vms371197‐sup‐0001‐S1.xlsx.

## Data Availability

The data that support the findings of this study are available from the corresponding author upon reasonable request.
